# Genomic correlation, shared loci, and causal link between obesity and diabetic microvascular complications: A genome-wide pleiotropic analysis

**DOI:** 10.17305/bb.2025.11897

**Published:** 2025-04-07

**Authors:** Wei Zhang, Qinghua Zhang, Yan Luo, Leilei Ma, Xuejun Wang, Qiao Zheng, Xiaotian Zhang, Shentao Wu, Zhan Li, Yingfei Bi

**Affiliations:** 1 Department of Cardiology, First Teaching Hospital of Tianjin University of Traditional Chinese Medicine, National Clinical Research Center for Chinese Medicine Acupuncture and Moxibustion, Tianjin, China; 2 First Teaching Hospital of Tianjin University of Traditional Chinese Medicine, National Clinical Research Center for Chinese Medicine Acupuncture and Moxibustion, Tianjin, China; 3 The Fourth Clinical Medical College of Guangzhou University of Chinese Medicine, Shenzhen, China; 4 Chiba Cancer Center Research Institute, Chiba, Japan

**Keywords:** Shared genetic architecture, obesity, diabetic microvascular complications, global genetic correlation

## Abstract

Observational studies have identified a connection between obesity and microvascular complications in diabetes, yet the genetic contributions to their co-occurrence remain incompletely understood. Our research aims to explore the shared genetic architecture underlying both conditions. We employed linkage disequilibrium score regression (LDSC) and Local Analysis of [co]Variant Association (LAVA) to assess genetic correlations between obesity and diabetic microvascular complications. To validate shared genetic regions, we conducted pleiotropic analysis under the composite null hypothesis (PLACO), functional mapping and annotation (FUMA), and colocalization analysis. Additionally, we applied Multimarker Analysis of GenoMic Annotation (MAGMA), Summary-based Mendelian Randomization (MR), multi-trait colocalization, and enrichment analysis to identify potential functional genes and pathways. Finally, MR and latent causal variable (LCV) analysis were used to clarify causal and pleiotropic relationships across trait pairs. Among 21 trait pairs analyzed, 15 exhibited significant global genetic correlations, and 97 regions showed significant local correlations. PLACO identified 3659–20,489 potentially pleiotropic single nucleotide polymorphisms (SNPs) across 15 trait pairs, with 828 lead SNPs detected via FUMA. Colocalization analysis confirmed 52 shared loci. Gene-based analysis identified seven unique candidate pleiotropic genes, with ribosomal protein S26 (*RPS26*) emerging as the strongest shared gene. MR and LCV analyses supported a causal relationship between BMI and diabetic kidney disease (DKD). Our findings highlight a shared genetic basis linking obesity with diabetic microvascular complications. These insights offer potential pathways for understanding the mechanisms driving their comorbidity and may inform more integrated therapeutic approaches.

## Introduction

Diabetic microvascular complications are a significant cause of mortality in patients with diabetes [1]. Diabetic kidney disease (DKD), diabetic retinopathy (DR), and diabetic neuropathy (DN) are hallmark manifestations of these complications and frequently co-occur in affected individuals [2]. DKD can rapidly progress to end-stage kidney disease, and currently, there are no specific and effective drug treatments available to halt its progression [3]. Additionally, DR can lead to vision loss, significantly impairing patients’ quality of life [4]. DN—which includes both peripheral and autonomic forms—is a common complication among individuals with diabetes. It often presents with a “stocking and glove” distribution of sensory symptoms and may also affect vital organs, such as the heart, kidneys, and bladder [5]. Obesity plays a central role in the development of diabetes mellitus and significantly exacerbates both its microvascular and macrovascular complications [6]. Weight management is therefore a fundamental strategy for reducing the risk of microvascular complications in people with diabetes [7], and optimizing lipid profiles can further enhance these protective effects [8, 9]. Research suggests that a combination of lifestyle interventions and early pharmacological treatment can help preserve microvascular function in individuals with prediabetes [10]. Moreover, Roux-en-Y gastric bypass (RYGB) surgery has been shown to reduce proteinuria in patients with type 2 diabetes mellitus (T2DM) and obesity who are in the early stages of chronic kidney disease [11]. As a result, the American Diabetes Association (ADA) emphasizes the importance of behavioral modifications, pharmacotherapy, and surgical interventions for achieving weight loss and reducing obesity-related complications in patients with T2DM [12]. In conclusion, while the strong link between obesity and diabetic microvascular complications is well recognized, the underlying mechanisms remain incompletely understood and warrant further investigation. Genetic studies provide compelling evidence that weight management is an effective strategy for preventing these complications—independent of glucose control—offering a novel perspective to explore potential shared genetic pathways between obesity and diabetic microvascular complications [13].

Obesity and diabetic microvascular complications are believed to share a significant genetic basis. Large-scale genome-wide association studies (GWAS) have identified numerous genetic markers linked to both conditions, lending strong support to this perspective [14, 15]. The field of research exploring the interplay between genetics and disease has made considerable progress. For example, genetic associations have been established between body mass index (BMI) and polycystic ovary syndrome [16], as well as between multiple sclerosis and inflammatory bowel disease [17]. However, the genetic connection between obesity and diabetic microvascular complications remains relatively underexplored. Despite the complex and multifactorial nature of these diseases, genetic factors are known to play a critical role in both their onset and progression. A more detailed investigation of specific genetic loci underlying these correlations is essential for deepening our understanding of their genetic foundations and for developing more targeted strategies for prevention and treatment [18].

**Figure 1.**
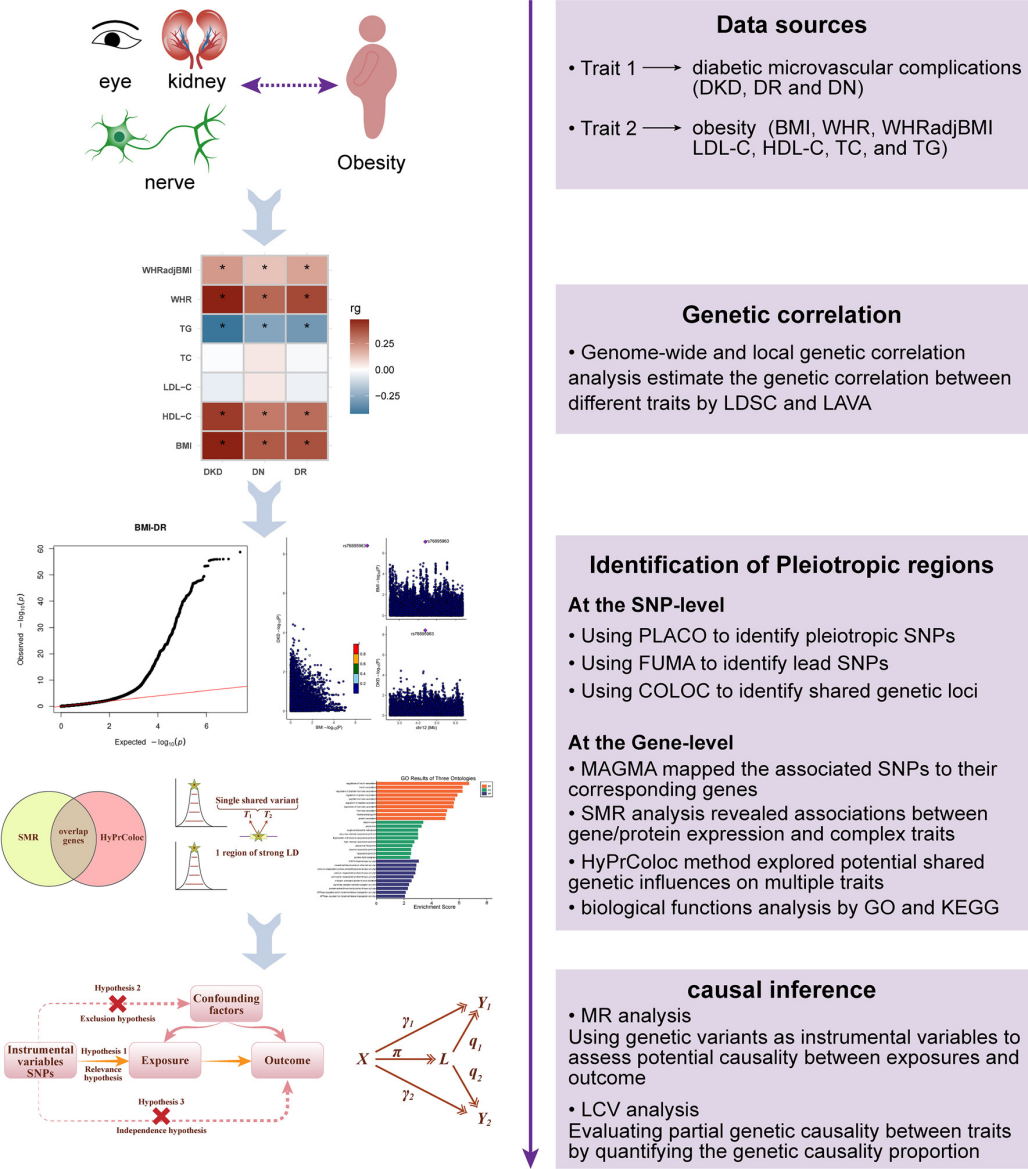
**Overview of statistical analyses performed in the study.** GWAS summary statistics on 7 obesity-related traits and diabetic microvascular complications were retrieved. First, we investigated the global and local genetic correlations among each pair of traits. Subsequently, we used a series of comprehensive approaches to identify pleiotropic variants and genes. Finally, the potential causality or pleiotropy behind these diseases were further explored. LDSC: Linkage disequilibrium score; LAVA: Local Analysis of [co]Variant Association; PLACO: Pleiotropic analysis under composite null hypothesis; FUMA: Functional mapping and annotation of genetic associations; MAGMA: Multimarker Analysis of GenoMic Annotation; SMR: Summary-based Mendelian Randomization; MR: Mendelian Randomization; LCV: Latent causal variable; GO: Gene Ontology; KEGG: Kyoto Encyclopedia of Genes and Genomes; DKD: Diabetic kidney disease; DR: Diabetic retinopathy; DN: Diabetic neuropathy; BMI: Body mass index; WHR: Waist-to-hip ratio; WHRadjBMI: Waist-to-hip ratio adjusted for body mass index; HDL-C: High-density lipoprotein cholesterol; TG: Triglycerides.

In this comprehensive genome-wide study of shared genetics, we conducted an extensive comparative analysis of seven obesity-related traits—BMI, waist-to-hip ratio (WHR), WHR adjusted for BMI (WHRadjBMI), low-density lipoprotein cholesterol (LDL-C), high-density lipoprotein cholesterol (HDL-C), total cholesterol (TC), and triglycerides (TG)—alongside three types of diabetic microvascular complications: DKD, DR, and DN. Our primary objective was to identify potential shared genetic factors between these traits using a range of statistical genetics methodologies. We began by examining global and local genetic correlations between each pair of traits. This was followed by the application of a comprehensive suite of analytic tools—including pleiotropic analysis under the composite null hypothesis (PLACO), functional mapping and annotation (FUMA), colocalization analysis, Multimarker Analysis of GenoMic Annotation (MAGMA), summary data–based Mendelian randomization (SMR), and multi-trait colocalization—to detect pleiotropic variants and genes. Lastly, we explored potential causal relationships or pleiotropy underlying these traits using Mendelian randomization (MR) and the latent causal variable (LCV) approach. A flowchart outlining the main analytical steps is presented in Figure 1.

## Materials and methods

### GWAS data sets

The obesity-related data in this study were primarily derived from two main sources. The first dataset included information on BMI, WHR, and WHRadjBMI, obtained from a large-scale meta-analysis combining data from the UK Biobank and the GIANT consortium [19]. This study represents the most extensive genome-wide association analysis of obesity to date, incorporating approximately 700,000 individuals of European ancestry [19]. The second dataset consisted of summary statistics from the Global Lipids Genetics Consortium, which included 1,654,960 participants from five distinct genetic ancestry groups [20]. For our analysis, we specifically used GWAS summary data for four lipid traits—LDL-C, HDL-C, TC, and TG—limited to participants of European descent. These GWAS analyses were adjusted for age, age squared, sex, principal components, and study-specific covariates to account for potential confounding factors [20]. The genetic data for diabetic microvascular complications were sourced from the Finnish Biobank Alliance (FinnGen), version 9. The GWAS cohorts for DKD, DN, and DR comprised 4,111, 2,843, and 10,413 cases, respectively, with corresponding control groups of 308,539, 271,817, and 308,633 individuals [21]. These complications were identified using ICD-10 codes. All datasets utilized in this study are publicly available, as detailed in Table S1.

### Statistical analysis

#### Identification of genetic correlations

Linkage disequilibrium score regression (LDSC) is a widely used method for estimating genetic correlations between traits using GWAS summary statistics. In this study, we employed LDSC to assess the genetic correlations between obesity-related traits and diabetic microvascular complications [22]. Prior to analysis, we preprocessed the data by filtering single nucleotide polymorphisms (SNPs) using the HapMap3 reference panel, ensuring high-quality and consistent SNP selection. LDSC estimates genetic correlation (*r_g_*) on a scale from −1 to 1. An absolute *r_g_* value closer to one indicates a strong genetic correlation, suggesting a substantial shared genetic basis between the traits, while values closer to 0 imply a weaker correlation. In general, an *r_g_* greater than 0.1 is considered indicative of a meaningful genetic relationship. To determine statistical significance, we applied a Bonferroni correction for multiple comparisons, setting the threshold at *P* < 0.002 (0.05/21). While LDSC assesses genome-wide correlations, it may overlook regional associations. To address this limitation, we also applied the Local Analysis of [co]Variant Association (LAVA) method, which enables investigation of local genetic correlations within specific genomic regions [23]. The LAVA approach partitions the human genome into 2495 independent segments, each approximately 1 Mb in size, allowing for more precise detection of shared genetic architecture at the regional level. For this analysis, we applied a Bonferroni-corrected significance threshold of *P* < 0.00002 (0.05/2495).

### Identification of pleiotropic regions

PLACO is a novel statistical method designed to identify pleiotropic SNPs shared between two traits [24]. Its core concept involves testing each SNP against a composite null hypothesis that assumes the SNP is associated with either one trait or neither. By using the product of two sets of Z-statistics as input, PLACO decomposes the composite null into three sub-scenarios, alongside an alternative hypothesis that represents true pleiotropic association. Unlike traditional SNP-based association analyses, PLACO’s composite hypothesis framework reduces false positives that can arise from imbalanced SNP effects between traits. Following PLACO analysis, SNPs with *P* < 5 × 10^−8^ are considered significant pleiotropic variants, suggesting potential influence across multiple traits. However, PLACO does not directly identify which specific SNPs contribute most to the observed linkage disequilibrium (LD). To refine our findings, we used FUMA, which incorporates LD information and additional genomic data to identify lead SNPs from the PLACO results. This analysis applied an LD threshold of R2 < 0.1 within a 1 Mb window [25]. To further investigate the biological relevance of these lead SNPs, we performed a comprehensive colocalization analysis on the loci containing them. Colocalization analysis evaluates the likelihood that observed associations for two traits arise from the same causal variant, based on five mutually exclusive hypotheses: H0 (no association with either trait), H1 and H2 (association with only one of the two traits), H3 (association with both traits, but due to distinct causal variants), and H4 (association with both traits due to a shared causal variant). A posterior probability for H4 (PPH4) exceeding 0.95 indicates strong evidence of a shared genetic basis at that locus [26].

### Functional annotation and enrichment analysis

MAGMA is a widely used tool for gene-based analysis of GWAS data. It aggregates the associations of multiple SNPs within defined gene regions while accounting for LD between SNPs, enabling efficient mapping of associated variants to their corresponding genes and laying the groundwork for comprehensive genome annotation. In this study, we followed the default settings in the FUMA software and incorporated SNP *P* values derived from the earlier PLACO analysis to perform MAGMA-based gene analysis [27]. Additionally, we employed SMR, a method that integrates GWAS data with functional biological information—such as gene expression and protein abundance—to examine associations between genes or proteins and target traits [28]. Specifically, we selected SNPs from eight different GWAS datasets representing traits, such as BMI, WHR, WHRadjBMI, TG, HDL-C, DKD, DR, and DN as instrumental variables. These SNPs were then jointly analyzed with expression quantitative trait loci (eQTL) data from blood, kidney, and pancreas tissues, as well as protein quantitative trait loci (pQTL) data from blood, in order to investigate their functional roles across different tissues [29, 30]. To evaluate whether the observed relationships between QTLs and traits were influenced by intergenic linkage effects or collinearity, we applied the heterogeneity in dependent instruments (HEIDI) test. We considered associations significant if they met both a Bonferroni-corrected *P* value threshold (calculated as 0.05 divided by the number of tests in each group) and a HEIDI *P* value greater than 0.05. To further assess the causality of SMR findings, we utilized the Hypothesis Prioritization for Multi-trait Colocalization (HyPrColoc) method. This approach identifies shared genetic influences contributing to multiple traits by prioritizing plausible causal configurations. HyPrColoc is capable of efficiently analyzing large numbers of traits and focuses on a small set of likely causal variants. We considered results with a PPH4 greater than 0.7 to be highly stringent and indicative of shared genetic architecture across traits [31]. Finally, to gain insight into the potential biological functions of genes identified through both colocalization and MAGMA analyses, we conducted Gene Ontology (GO) and Kyoto Encyclopedia of Genes and Genomes (KEGG) pathway enrichment analyses. These were performed using the clusterProfiler and pathview R packages, with significance defined as *P* < 0.05 [32, 33].

### Assessment causal and pleiotropy relationship

MR and LCV analyses were employed to investigate causal and pleiotropic relationships between various trait pairs, offering complementary perspectives. MR uses genetic variants as instrumental variables to assess potential causal relationships between exposures—such as environmental factors or behaviors—and disease outcomes. The underlying premise is that if a genetic variant is strongly associated with an exposure, and the exposure is in turn associated with disease risk, then the variant may exert a causal effect on disease susceptibility [34, 35]. To assess the robustness of MR findings, we used Cochran’s *Q* test to detect heterogeneity in the individual causal effects and applied MR-Egger’s intercept test to evaluate the presence of horizontal pleiotropy. LCV offers a more nuanced framework for disentangling the causal structure underlying genetic correlations. It introduces an LCV that mediates genetic effects across traits. When a trait shows strong genetic correlation with this latent variable, it is inferred to exert a partial genetic causal effect on the other trait. LCV quantifies this relationship using the genetic causality proportion (GCP) metric: a GCP close to one suggests a predominant causal genetic influence, while a value near 0 indicates that pleiotropy plays a larger role. The sign of the GCP also indicates the direction of the causal effect. In this study, a GCP value above 0.7 was interpreted as evidence of a substantial genetic causal effect, suggesting that most of the observed genetic correlation was likely driven by causal mechanisms [35]. Together, MR and LCV provided a robust analytical framework for distinguishing between pleiotropic effects and true causal relationships, offering a more comprehensive understanding of the genetic architecture underlying complex diseases. To control for false positives due to multiple testing, we applied a Bonferroni correction.

## Results

### Overall and local genetic correlation

**Figure 2.**
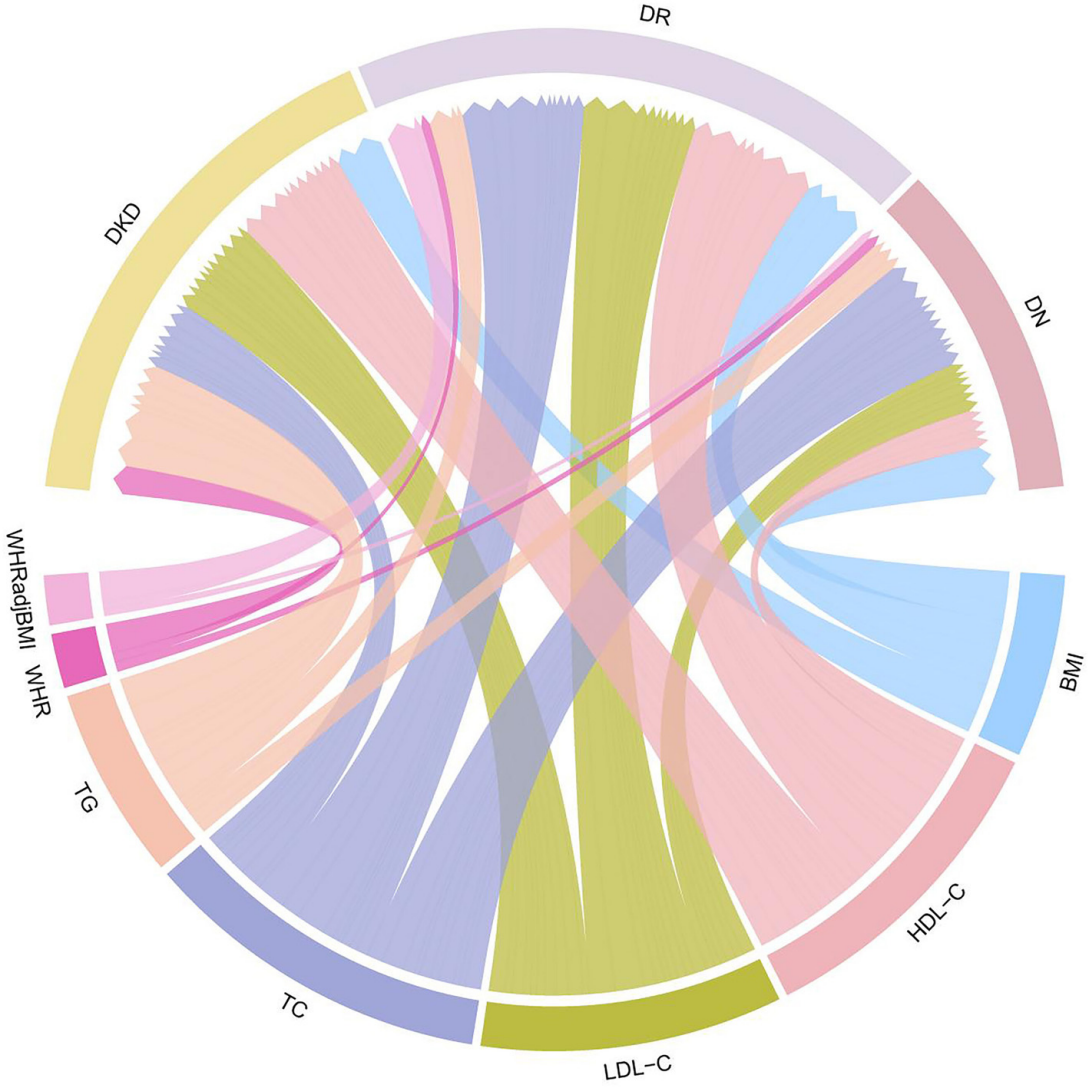
**Local genetic correlations between obesity-related traits and diabetic microvascular complications.** Distinct colors are used to represent different traits, while the width of the connecting bands (chords) reflects the strength of the relationships between genes. A wider band indicates a stronger correlation between traits, while a narrower band signifies a weaker correlation. Only correlations meeting *P* < 0.00002 (0.05/2495) are displayed. DKD: Diabetic kidney disease; DR: Diabetic retinopathy; DN: Diabetic neuropathy; BMI: Body mass index; WHR: Waist-to-hip ratio; WHRadjBMI: Waist-to-hip ratio adjusted for body mass index; HDL-C: High-density lipoprotein cholesterol; TG: Triglycerides.

The overall genetic correlation results indicate that BMI, WHR, WHRadjBMI, TG, and HDL-C exhibit moderate positive correlations with both diabetic microvascular complications. Among them, BMI and WHR display the strongest correlation with DKD (*r_g_* ═ 0.47, *P* ═ 4.725e-26; *r_g_* ═ 0.47, *P* ═ 4.40e-22). Additionally, TG demonstrates a negative genetic correlation with DKD, DR and DN (*r_g_* ═ –0.42, *P* ═ 8.062e-16; *r_g_* ═–0.31, *P* ═ 2.181e-14; *r_g_* ═ –0.27, *P* ═ 8.41e-09). However, TC demonstrates nearly negligible correlation with DKD, DR and DN (*r_g_* ═ –0.01, *P* ═ 0.704; *r_g_* ═ –0.02, *P* ═ 0.547), similar to the results observed for LDL-C (*r_g_* ═ –0.05, *P* ═ 0.301; *r_g_* ═–0.04, *P* ═ 0.358; *r_g_* ═ 0.05, *P* ═ 0.257). In summary, out of the 21 trait pairs analyzed, only 15 showed significant global genetic correlations (Table 1, Figure S1, and Table S2). The LAVA analysis identified a total of 97 significant local genetic correlations across these trait pairs. Notably, LDL-C and HDL-C exhibited the highest number of significant local correlations with DKD, each contributing 10 regions. Interestingly, these findings contrast with the global genetic correlation analysis, which did not detect a significant relationship between LDL-C and either DR or DKD. Similarly, a positive global genetic correlation was observed between WHRadjBMI and DKD; however, no corresponding local genetic correlation was found between these traits in the LAVA analysis. Surprisingly, despite the weak or negligible global genetic correlations between TC and DKD, DR, or DN, LAVA revealed 7, 9, and 9 significant local correlations for these trait pairs, respectively (Figure 2 and Table S3).

**Table 1:** Genome-wide genetic correlation between diabetic microvascular complications and obesity-related traits

**Trait1**	**Trait2**	**r_g_**	**SE**	***P* value**
DKD	BMI	0.47	0.0447	4.73e-26
DKD	WHR	0.47	0.0488	4.40e-22
DKD	WHRadjBMI	0.22	0.0437	5.07e-07
DKD	LDL-C	−0.05	0.0447	3.01e-01
DKD	HDL-C	0.43	0.0473	2.17e-19
DKD	TC	−0.01	0.0378	7.04e-01
DKD	TG	−0.42	0.0519	8.06e-16
DR	BMI	0.38	0.0348	1.28e-27
DR	WHR	0.40	0.0347	8.54e-31
DR	WHRadjBMI	0.20	0.0321	6.72e-10
DR	LDL-C	−0.04	0.0382	3.58e-01
DR	HDL-C	0.32	0.0412	6.19e-15
DR	TC	−0.02	0.0324	5.47e-01
DR	TG	−0.31	0.0402	2.18e-14
DN	BMI	0.36	0.0487	1.45e-13
DN	WHR	0.33	0.0425	5.01e-15
DN	WHRadjBMI	0.13	0.0368	3.01e-04
DN	LDL-C	0.05	0.0453	2.57e-01
DN	HDL-C	0.29	0.0475	7.47e-10
DN	TC	0.05	0.0403	1.97e-01
DN	TG	−0.27	0.0464	8.41e-09

r_g_: Genetic correlation; SE: Standard error; BMI: Body mass index; WHR: Waist-to-hip ratio; WHRadjBMI: Waist-to-hip ratio adjusted for body mass index; LDL-C: Low-density lipoproteins cholesterol; HDL-C: High-density lipoprotein cholesterol; TC: Total cholesterol; TG: Triglycerides; DKD: Diabetic kidney disease; DR: Diabetic retinopathy; DN: Diabetic neuropathy.

### Pleiotropic regions validation

Within the scope of these 15 distinct traits, PLACO analysis revealed a spectrum of potential pleiotropic SNPs ranging from 3659 to 20,489, with a total of 37,738 unique SNPs (Figure S2). Subsequent meticulous scrutiny using FUMA identified a subset of 828 independent SNPs, representing instances of pleiotropy. Among these, the HDL-C and DR trait pair exhibited the highest abundance, with 103 lead SNPs, while the TG-DN trait pair displayed the lowest count, with 25 lead SNPs. Additionally, among these lead SNPs, rs429358 concurrently influenced 11 pairs of traits, while rs7903146 impacted seven pairs of traits (Table S4). According to ANNOVAR’s categorization, as a facet of FUMA’s capabilities, among the 828 lead SNPs, 41.7% were found to be intronic variants, while 34.6% were intergenic variants. Exonic variants, which included eight noncoding RNA exonic variants, made up only 5.4% of the total. Furthermore, there were 20 UTR3 variants (2.4%) and six UTR5 variants (0.7%) (Table S4). Subsequent colocalization analysis unveiled 52 loci with strong colocalization signals, all surpassing a PPH4 threshold of 0.95. Notably, among these loci, 10 were associated with DKD, 40 with DR, and two with DN. Within these loci, rs10938397 emerged as a pivotal candidate locus linked to DR, exhibiting concurrent evidence of associations with BMI, HDL-C, and WHR. Moreover, another SNP, rs429358, demonstrated colocalization evidence in both DKD and DR, displaying significant correlations with HDL-C, WHR, and WHRadjBMI traits. Similarly, rs7144011 showed a significant association with DR, and further analysis revealed its associations with a range of obesity-related characteristics, including BMI, HDL-C, TG, and WHR (Table 2, Figure S3, and Table S5).

**Table 2:** 52 colocalized loci identified by colocalization analysis

**Trait pair**	**Lead SNP**	**CHR**	Locus boundary*	**PPH4**		**Trait pair**	**Lead SNP**	**CHR**	Locus boundary*	**PP.H4**
BMI-DKD	rs7903146	10	114722134-114818754	0.991		TG-DR	rs9379084	6	7231843-7231843	0.980
BMI-DKD	rs76895963	12	4384844-4384844	1.000		TG-DR	rs7451008	6	20641336-20727570	0.982
BMI-DR	rs10938397	4	45068929-45193147	0.996		TG-DR	rs1708302	7	28142088-28209953	0.967
BMI-DR	rs849135	7	28142088-28209953	0.977		TG-DR	rs3802177	8	118184783-118220270	0.957
BMI-DR	rs6602411	10	10255003-10264200	0.993		TG-DR	rs10811661	9	22132076-22136489	0.989
BMI-DR	rs7903146	10	114729482-114867427	0.977		TG-DR	rs7903146	10	114729482-114818754	0.994
BMI-DR	rs1557765	11	17368013-17421886	0.987		TG-DR	rs10765572	11	92668975-92708710	0.957
BMI-DR	rs76895963	12	4384844-4384844	1.000		TG-DR	rs76895963	12	4384844-4384844	1.000
BMI-DR	rs12885454	14	29680331-29777492	0.965		TG-DR	rs7144011	14	79833494-79945162	0.979
BMI-DR	rs7144011	14	79703248-79945162	0.980		TG-DR	rs483082	19	45232161-45524119	0.967
HDL-C-DKD	rs429358	19	45337918-45523583	0.999		WHR-DKD	rs429358	19	45392254-45424351	0.999
HDL-C-DR	rs11708067	3	122936084-123131254	0.957		WHR-DR	rs11705729	3	185488303-185538006	0.955
HDL-C-DR	rs10938397	4	45164637-45187622	0.996		WHR-DR	rs10938397	4	45164637-45187622	0.995
HDL-C-DR	rs1574285	9	4282536-4296430	0.980		WHR-DR	rs1513272	7	28142088-28209953	0.986
HDL-C-DR	rs11171739	12	56368078-56584247	0.984		WHR-DR	rs7144011	14	79833494-79945162	0.984
HDL-C-DR	rs3184504	12	111662984-113218868	0.995		WHR-DR	rs9923544	16	53797908-53848561	0.951
HDL-C-DR	rs7144011	14	79833494-79945162	0.979		WHR-DR	rs429358	19	45386467-45428234	1.000
HDL-C-DR	rs151249695	15	38909425-38909425	0.997		WHRadjBMI-DKD	rs9356744	6	20635719-20727570	0.962
HDL-C-DR	rs429358	19	45324138-45623467	1.000		WHRadjBMI-DKD	rs429358	19	45392254-45424351	0.998
TG-DKD	rs7766070	6	20652717-20703952	0.974		WHRadjBMI-DR	rs112256201	3	50599511-50724724	0.959
TG-DKD	rs7903146	10	114729482-114817009	0.996		WHRadjBMI-DR	rs4686696	3	185488303-185538006	0.959
TG-DKD	rs76895963	12	4328521-4384844	1,000		WHRadjBMI-DR	rs1513272	7	28142088-28256240	0.981
TG-DKD	rs695399	22	29889324-30082569	0.951		WHRadjBMI-DR	rs1002226	11	17368013-17421886	0.980
TG-DR	rs28408152	3	115063640-115102814	0.966		WHRadjBMI-DR	rs7310615	12	111826477-112906415	0.961
TG-DR	rs11716713	3	185488303-185538006	0.959		WHRadjBMI-DR	rs429358	19	45388500-45424351	0.999
BMI-DN	rs7903146	10	114749734-114817009	0.970		TG-DN	rs7903146	10	114749734-114817009	0.986

*Locus boundary displays the region (start-end) defined by FUMA analysis. SNP: Single nucleotide polymorphism; CHR: Chromosome; PPH4: Posterior probability of H4; BMI: Body mass index; WHR: Waist-to-hip ratio; WHRadjBMI: Waist-to-hip ratio adjusted for body mass index; HDL-C: High-density lipoprotein cholesterol; TG: Triglycerides; DKD: Diabetic kidney disease; DR: Diabetic retinopathy; DN: Diabetic neuropathy.

### Shared gene function and enrichment analysis

**Figure 3.**
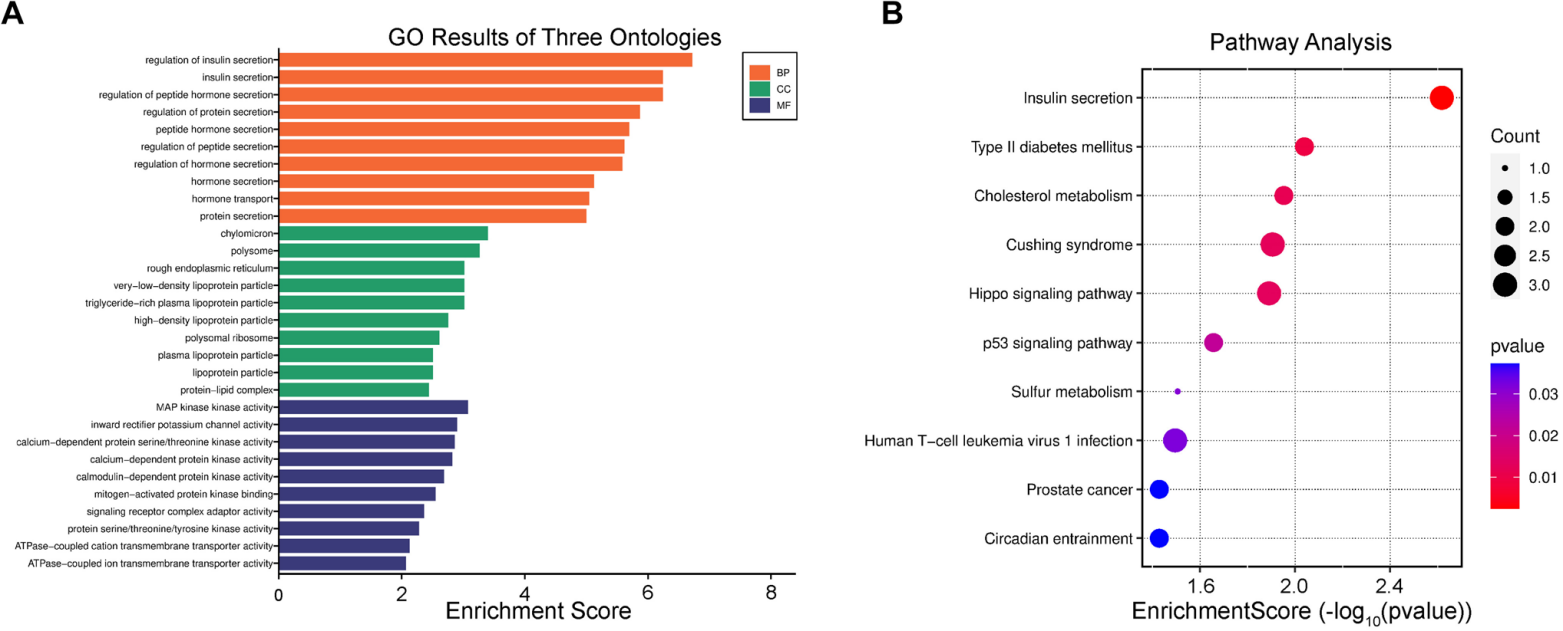
**The GO and KEGG enrichment analysis of genes in the region of the colocalization analysis results.** (A) Different colors are used to represent the three main categories of Gene Ontology terms: BP, CC, and MF. Each bar in the chart corresponds to enrichment score of the GO term within the respective category. (B) The x-axis represents enrichment score, and y-axis represents different biological pathways. The size of circle represents gene count, with larger circles indicating more genes and smaller circles indicating fewer genes. Circle colors indicate *P* values, with blue representing higher *P* values (less significant) and red representing lower *P* values (more significant). GO: Gene Ontology; KEGG: Kyoto Encyclopedia of Genes and Genomes; CC: Cellular component; BP: Biological process; MF: Molecular function.

In this study, we employed various analytical methods to delve into potential shared genetic influences among multiple traits. Firstly, we identified a total of 4164 pleiotropic genes through MAGMA analysis, of which 88 overlapped with genes in the region of the colocalization analysis results (Table S6). Notably, several genes, such as APOE, PVRL2, and TOMM40 exhibited significance across all seven pairs of traits, followed by APOC1, which was implicated in five trait pairs. Subsequently, we conducted SMR analyses on GWAS data, eQTL data (including whole blood, kidney, and pancreas), and whole-blood pQTL data, leading to the discovery of 879 shared genes and proteins. In the eQTL analysis, we found 348, 91, and 259 shared genes in the non-MHC regions of whole blood, kidney, and pancreas, respectively. Particularly in kidney tissue, C4A was identified as a shared gene across multiple traits, encompassing WHR and DR/DKD/DN, TG and DR/DKD/DN, as well as BMI and DR/DKD/DN. Additionally, we observed the presence of XXbac-BPG254F23.7 as shared between HDL-C and DR/DKD/DN, and intriguingly, ribosomal protein S26 (RPS26) emerged as a shared gene not only between TG and DR but also between HDL-C and DR/DKD. Notably, it’s important to highlight that previous colocalization analyses focusing on HDL-C and DR had already pinpointed RPS26 as one of the shared genetic variants. Similarly, in pancreas tissue, RPS26 was identified as a shared gene between TG and DR. However, no shared genes were identified in blood tissue (Table S7). Using pQTL data, MANBA (associated with the corresponding protein) was exclusively found in the BMI-DR pair (Table S8). Lastly, in the multi-trait colocalization analysis, we discovered a series of shared genes across different trait pairs, including JAZF1, NCR3LG1, RP1-239B22.5, SUOX, ZBTB20, IKZF4, and NIPSNAP1 (Table S9). However, the most surprising finding was the identification of RPS26 between HDL-C and DR, as well as in the eQTL (kidney) analyses. This result was consistently validated across the multi-trait colocalization analysis, SMR analysis, and previous colocalization analyses. Moreover, all three analytical methods supported the lead SNP: rs11171739. In the GO enrichment analysis, we identified 311 enriched biological process (BP) pathways, 33 enriched cellular component (CC) pathways, and 57 enriched molecular function (MF) pathways. For example, the “regulation of insulin secretion” (GO:0050796, *P* ═ 1.89e-07) pathway exhibited the most significant enrichment in BP, while “chylomicron” (GO:0042627, *P* ═ 3.98e-04) and “MAP kinase kinase activity” (GO:0004708, *P* ═ 8.39e-04) showed significant enrichment in CC and MF, respectively (Figure 3A). Additionally, we conducted KEGG pathway enrichment analysis and identified 13 significantly enriched pathways, with “Insulin secretion” (KEGG: hsa04911, *P* ═ 2.41-3e) displaying significant enrichment (Figure 3B).

### Causal and pleiotropy inference

**Figure 4.**
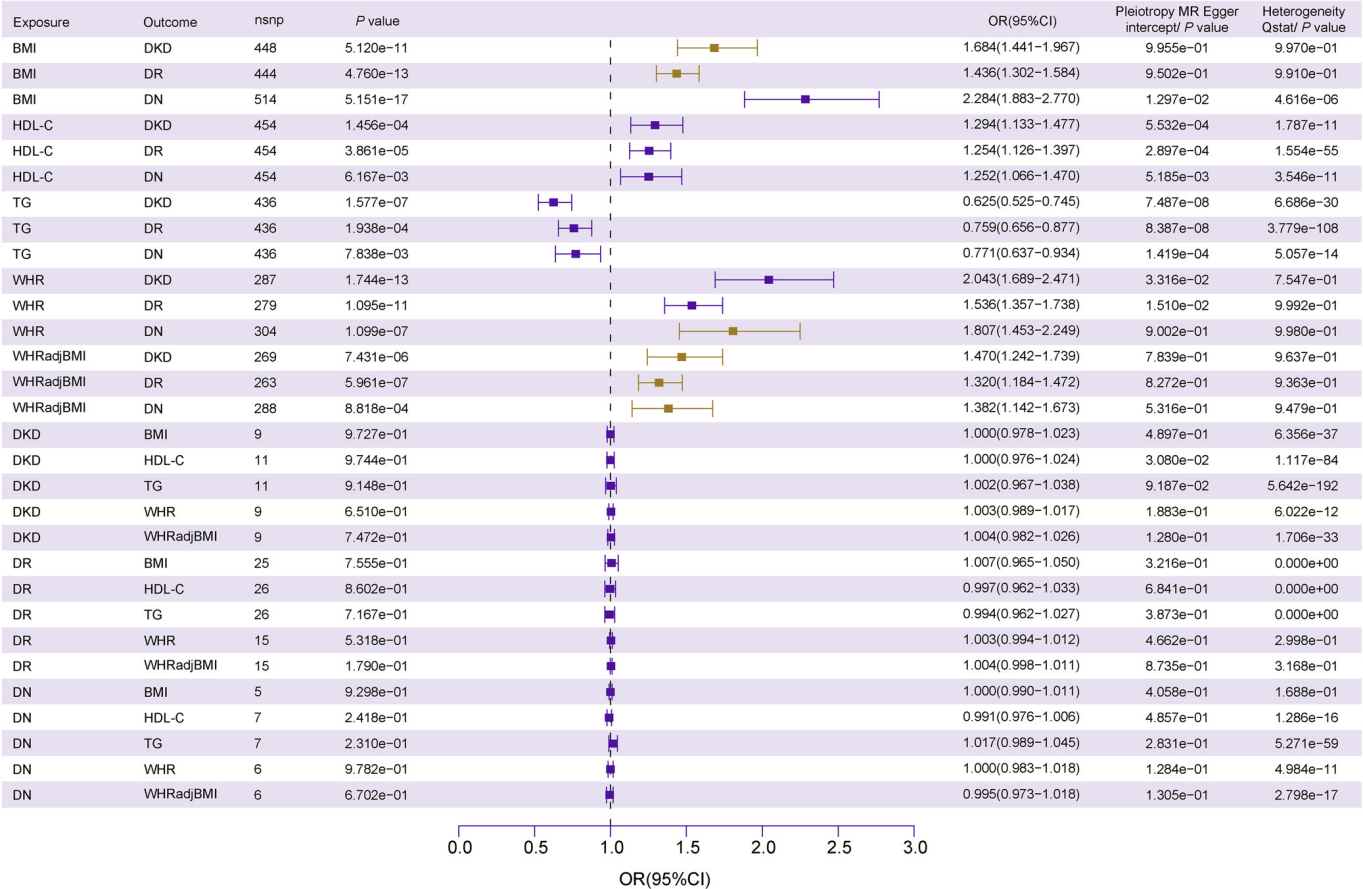
**Summary of bi-directional MR analyses between obesity-related traits and diabetic microvascular complications.** Error bars represent the 95% confidence intervals for the associated MR estimates. The primary method for *P* value calculation is the IVW method. The MR-Egger intercept and Cochrane’s *Q* test were used to assess pleiotropy and heterogeneity. A significant MR-Egger intercept (*P* < 0.05) suggests pleiotropic effects, while a significant Cochrane’s *Q* test (*P* < 0.05) indicates heterogeneity. DKD: Diabetic kidney disease; DR: Diabetic retinopathy; DN: Diabetic neuropathy; BMI: Body mass index; WHR: Waist-to-hip ratio; WHRadjBMI: Waist-to-hip ratio adjusted for body mass index; HDL-C: High-density lipoprotein cholesterol; TG: Triglycerides; MR: Mendelian randomization.

To delve into the causal relationships between diabetic microvascular complications (DKD, DR, and DN) and obesity-related traits (HDL-C, TG, BMI, WHRadjBMI, and WHR), we employed a bidirectional MR approach with the inverse variance weighted (IVW) method as the primary analytical tool. The results show clear causal relationships in only six pairs: BMI-DKD (*P*_IVW_ ═ 5.12e-11, OR ═ 1.68[1.44, 1.97]), BMI-DR (*P*_IVW_ ═ 4.76e-13, OR ═ 1.44[1.30, 1.58]), WHRadjBMI-DKD (*P*_IVW_ ═ 7.43e-06, OR ═ 1.47[1.24, 1.74]), WHRadjBMI-DR (*P*_IVW_ ═ 5.96e-07, OR ═ 1.32[1.18, 1.47], WHR-DN (*P*_IVW_ ═ 1.10e-07, 0R ═ 1.81[1.45, 2.25]), and WHRadjBMI-DN (*P*_IVW_ ═ 8.8e-04, 0R ═ 1.38[1.14, 1.67]). Additionally, all the aforementioned results have been subjected to heterogeneity (*P* > 0.05) and pleiotropy testing (*P* > 0.05). Notably, no evidence of reverse causation was observed among these factors (Figure 4 and Table S10). To fortify the integrity of our findings, we judiciously employed LCV. This rigorous approach reaffirmed the causal nexus between BMI and DKD (*P* ═ 1.55e-4, GCP ═ 0.75), substantiating the robustness of this association. In contrast to the MR findings, there is also a strong genetic causality between HDL-C and DN (*P* ═ 4.71e-14, GCP ═ 0.82) (Table S11).

## Discussion

To the best of our knowledge, this is the first comprehensive genome-wide study to investigate the pleiotropic associations underlying the co-occurrence of obesity and microvascular complications in diabetes. We employed a multifaceted array of statistical methods to rigorously assess genetic correlations, pleiotropic variants and loci, and to explore potential shared gene sets, causal relationships, and relevant biological pathways. These findings help mitigate confounding factors present in observational studies, shed light on the etiology and comorbidity patterns between obesity and diabetic microvascular complications, and ultimately contribute to simplifying disease prevention and management.

We investigated the genetic connections between obesity-related traits and microvascular complications in diabetes and found incomplete concordance between genome-wide signals and those from specific genomic regions. For example, while the overall correlation between TC/LDL-C and diabetic microvascular complications is generally weak at the global genome level, it becomes more pronounced within particular regions. This likely reflects the complex and heterogeneous genetic architecture of these traits, where multiple loci may exert effects in opposing directions or have small effect sizes. Such signals can cancel each other out in global analyses, resulting in diluted correlations that fall short of statistical significance. Some loci may carry both risk and protective alleles simultaneously, or involve distinct biological pathways with antagonistic effects on the phenotype. Local analytical approaches are especially valuable in this context, as they can disentangle these opposing effects and reveal region-specific associations that global methods overlook. This precision enhances the identification of biologically meaningful targets for mechanistic studies and provides insight into context-dependent genetic contributions that are otherwise masked in genome-wide analyses.

In our global genetic correlation analysis, we observed a notably strong genetic association between BMI and DKD, aligning well with findings from both MR and LCV analyses. Specifically, the GCP for the BMI-DKD relationship was 0.75, exceeding the commonly used threshold of 0.7. This suggests that the genetic link between BMI and DKD is largely driven by a causal relationship, with minimal influence from horizontal pleiotropy. Additionally, the positive GCP value, combined with MR results showing no evidence of reverse causality, supports a directional pathway: genetic factors influence BMI (trait 1), which in turn affects DKD (trait 2). These findings are consistent with epidemiological data. A comprehensive systematic review and meta-analysis of 20 cohort studies identified BMI as an independent risk factor for DKD. Specifically, each 5 kg/m^2^ increase in BMI was associated with a 16% higher risk of developing DKD [36]. This convergence of genetic and epidemiological evidence reinforces the notion of a causal relationship between BMI and DKD. Using LDSC, we also identified a significant genetic correlation between HDL-C and diabetic nephropathy, with supporting evidence from LCV analysis. However, MR did not detect a significant causal effect in this case. This discrepancy likely reflects methodological differences: MR depends on instrumental variables and assumes a proportional relationship between genetic effects and the exposure, while LCV models genetic correlations more flexibly and accounts for pleiotropy. LCV’s broader instrument assumptions and efficient use of sample data confer greater statistical power, especially in studies with limited sample sizes. Moreover, LCV can detect partial causal components that MR might miss by quantifying the genetic causality proportion—an advantage when examining complex genetic architectures.

In our investigation of the shared genetic architecture underlying complex traits, we conducted a comprehensive analysis of pleiotropy at both the SNP and gene levels. To identify pleiotropic SNPs associated with specific traits, we employed a rigorous, multi-step analytical pipeline. We began with an initial screening using PLACO, which enabled the detection of candidate pleiotropic SNPs and laid the groundwork for subsequent analyses. Next, we used FUMA to refine these results, identifying 828 lead SNPs while carefully removing those in LD. Colocalization analysis then confirmed 52 loci with strong supporting evidence. To further explore the functional relevance of these loci, we applied an integrative approach combining MAGMA, SMR, and multivariate colocalization, ultimately identifying 102 genes associated with the 52 pleiotropic loci, 48 of which were unique. Among these, RPS26, along with its lead SNP rs11171739, emerged as the most robustly supported pleiotropic risk gene. RPS26 encodes ribosomal protein S26, which plays a vital role in ribosome biogenesis by participating in the processing of pre-ribosomal RNA. Beyond its canonical function, RPS26 exerts significant regulatory control over the conformational stability and transcriptional activity of p53, a key transcription factor that mediates cellular stress responses and maintains genomic integrity. Experimental studies have shown that both overexpression and suppression of RPS26 can enhance p53 stabilization, leading to downstream cellular effects, such as cell cycle arrest and apoptosis [37]. In the context of diabetes mellitus, p53 plays a largely detrimental role, disrupting cellular function and metabolic regulation. Cytoplasmic accumulation of p53 in pancreatic β-cells has been linked to the inhibition of Parkin-mediated mitophagy, contributing to mitochondrial dysfunction and impaired insulin secretion [38]. In renal tubules, p53 impairs autophagy by inducing miR-214, which suppresses ULK1, a key autophagy-initiating kinase [39]. Additionally, recent studies suggest that O-GlcNAc–mediated regulation of p53 stability may contribute to hyperglycemia-induced cell death in retinal pericytes [40]. Moreover, RPS26 appears to regulate T-cell survival through a p53-dependent mechanism. Murine studies have revealed elevated expression of RPS26 in T lymphocytes, and its deletion results in peripheral T-cell instability and disrupted thymic T-cell development [41]. This observation is particularly relevant given the immune system’s central role in the pathogenesis of diabetes. p53 has also been implicated in obesity-related pathways, including lipid metabolism, energy balance, and hormone sensitivity [42–44], further suggesting a potential link between RPS26, p53, and metabolic disorders. Based on these findings, we hypothesize that RPS26 contributes to diabetic microvascular complications and obesity through mechanisms involving p53 activation and stabilization. To investigate this hypothesis, co-immunoprecipitation (co-IP) and p53-responsive luciferase reporter assays should be conducted to assess protein–protein interactions and transcriptional modulation. *In vivo* studies using RPS26 transgenic or knockout mice can help determine its physiological relevance through blood glucose monitoring, insulin sensitivity testing, and histological examination of diabetic complications. *In vitro*, cell models with RPS26 overexpression or knockdown can be established via transfection with overexpression plasmids or siRNA, respectively. Western blotting would then be used to evaluate p53 protein levels under different RPS26 expression states.

In addition to the pronounced pleiotropic effects observed for RPS26, several other genes identified through multivariate colocalization analysis also exhibit substantial genetic influence. However, it is important to note that these genes did not surpass the stringent significance threshold in the SMR analysis—primarily due to the strong correction pressure imposed by their extensive pleiotropy. Among them, JAZF1 (Zinc Finger Protein 1), which is predominantly expressed in pancreatic tissue, is recognized as a key regulator of glucose and lipid metabolism. It interacts with several critical pathways, including adenosine monophosphate (AMP), AMP-activated protein kinase (AMPK), and mitogen-activated protein kinase (MAPK), exerting anti-glycemic, anti-lipidemic, and anti-inflammatory effects [45, 46]. Similarly, ZBTB20, another zinc finger protein highly expressed in pancreatic β-cells, contributes to glucose homeostasis by repressing the transcription of Fructose-1,6-bisphosphatase 1 (FBP1) [47]. This regulatory function supports β-cell performance and overall glucose regulation. In addition to its role in β-cells, ZBTB20 also contributes to hepatic de novo lipogenesis (DNL), thereby playing an important role in systemic lipid metabolism. Nipsnap1 has been extensively studied for its role in mitophagy, particularly in the recruitment of autophagy-related proteins to the mitochondrial outer membrane. This function is crucial for maintaining mitochondrial quality control and has significant implications for diabetes and its complications [48, 49]. Under chronic cold exposure, impairment or inhibition of Nipsnap1 can compromise cellular DNL and mitochondrial lipid β-oxidation capacity, further affecting metabolic balance [50]. IKZF4 and SUOX have been identified as susceptibility loci for diabetes [51, 52]; however, their involvement in obesity remains underexplored, with limited and inconclusive findings to date. Conversely, NCR3LG1 has primarily been associated with various cancer types [53, 54], while RP1-239B22.5 has received minimal research attention—only one study to date has reported elevated expression in late-stage cancer [55]. The limited functional annotation of these latter genes suggests that further investigation is necessary to clarify their potential roles in metabolic disorders.

In enrichment analysis, the regulation of insulin hormone secretion is highlighted in both the BP domain of GO and in KEGG pathways. This suggests that maintaining blood glucose homeostasis is a key factor in mitigating microvascular complications in diabetes. Additionally, the analysis shows that enrichment in the CC category is primarily associated with lipid metabolism, further emphasizing the link between obesity and diabetic microvascular complications. MF enrichment spans several areas, including signal transduction, ion transport, protein kinase activity, calcium-dependent processes, ATPase coupling, and more. These signaling pathways are critical for processes, such as cell proliferation, differentiation, survival, and apoptosis. Dysregulation of these pathways can contribute to cellular dysfunction, metabolic disorders, impaired insulin secretion, and vascular abnormalities [56–58]. For example, the MAPK family—which includes extracellular signal-regulated kinases (ERKs), Jun N-terminal kinases (JNKs), and p38/SAPKs (stress-activated protein kinases)—plays pivotal roles in the development of these conditions through mechanisms, such as promoting inflammation, disrupting insulin signaling, altering lipid metabolism, and impairing pancreatic islet function [59–61].

While our study has yielded important findings, several limitations should be acknowledged. First, although we utilized large-scale GWAS data for obesity-related traits, the available data for DKD, DR, and DN remain limited, which may affect the comprehensiveness of our findings. Second, all data sources were derived from individuals of European descent, potentially limiting the generalizability of our results to other populations. Future studies should aim to include a more diverse range of ancestries to better capture the genetic architecture of the traits under investigation. Lastly, to gain deeper insights into the functional and mechanistic roles of shared risk genes in the microvasculature associated with diabetes and obesity, further *in vitro* and *in vivo* studies are needed. These should account for tissue specificity, including but not limited to whole blood, kidney, and pancreas.

## Conclusion

In summary, our study identified significant genetic correlations between obesity and microvascular complications in diabetes, successfully pinpointing shared risk SNPs and genes—most notably, RPS26, which showed the strongest genetic association. We also explored causal and pleiotropic relationships in depth, offering valuable insights into the genetic mechanisms underlying these traits. These findings lay a strong foundation for future research into the pathogenesis and potential therapeutic strategies for these conditions.

## Supplemental data

Supplemental data are available at the following link: https://www.bjbms.org/ojs/index.php/bjbms/article/view/11897/3815.

## Data Availability

GWAS summary statistics for obesity: http://csg.sph.umich.edu/willer/public/glgc-lipids2021/ and https://zenodo.org/record/1251813∖#.ZGCbP7JBztU GWAS summary statistics for diabetic microvascular complications: https://www.finngen.fi/en/access_results. The pQTL summary data can be found at http://nilanjanchatterjeelab.org/pwas/ The codes used in this study can be found at: LDSC: https://github.com/bulik/ldsc. LAVA: https://github.com/josefin-werme/LAVA PLACO: https://github.com/RayDebashree/PLACO. COLOC: https://github.com/chr1swallace/coloc. SMR: https://cnsgenomics.com/software/smr/∖#Overview. FUMA: https://fuma.ctglab.nl/celltype/. TwoSampleMR: https://mrcieu.github.io/TwoSampleMR/. LCV: https://github.com/lukejoconnor/LCV. All the data and code are accessible in public databases and open for public access. Further inquiries can be directed to the corresponding author.
